# Inhibition of NF-κB Signaling Pathway by Resveratrol Improves Spinal Cord Injury

**DOI:** 10.3389/fnins.2018.00690

**Published:** 2018-10-04

**Authors:** Luyao Xu, Benson O. A. Botchway, Songou Zhang, Jingying Zhou, Xuehong Liu

**Affiliations:** ^1^Department of Histology and Embryology, Medical College, Shaoxing University, Shaoxing, China; ^2^Institute of Neuroscience, Zhejiang University School of Medicine, Hangzhou, China

**Keywords:** spinal cord injury, resveratrol, NF-κB signaling pathway, inflammation, apoptosis

## Abstract

Spinal cord injury (SCI) can have a significant impact on an individual’s life. Herein, we discuss how resveratrol improves SCI by inhibiting nuclear factor kappa-light-chain-enhancer of activated B cells (NF-κB) signaling pathway. Evidences show resveratrol suppresses NF-κB signaling pathway to exert its beneficial effects on various diseases. NF-κB signaling pathway plays a significant role in the pathophysiological mechanisms of SCI including increase in inflammation, augmentation of damage caused by free radicals and lipid peroxidation as well as facilitation of apoptosis and axonal demyelination. We also discuss mechanisms between resveratrol and NF-κB signaling pathway in the wake of SCI, which can be potential targets for resveratrol to treat SCI.

## Introduction

Spinal cord injury (SCI) is a severe complication of the spine, leading to severe dysfunction below injured segment. SCI puts pressure on a country’s health services whiles also burdening an affected individual’s family and society. The International Spinal Cord Society Prevention Committee estimates global-incident rate to be 23 traumatic SCI cases per million (Lee et al., 2014). SCI is categorized into primary injury and secondary injury phases on the basis of its pathophysiology. Although primary injury is transient and can be caused by a mechanical force, it, nevertheless, creates a basic environment for succeeding pathological events. Secondary injury includes a chain of pathological events such as apoptosis, inflammatory response, and excitotoxicity (Silva et al., 2014). SCI can result in different spinal cord dysfunctions and induce complications in other organs such as the lung (Jiang et al., 2016). As primary injury phase is transient, SCI treatment could focus on the secondary injury phase. With that said, there is presently no effective SCI treatment.

Several researches have demonstrated NF-κB to be a crucial mediator of cellular responses to numerous physiological stimuli. NF-κB signaling pathway has been shown to participate in mediating SCI pathophysiology, subsequently playing a pivotal role in repairing SCI. Inhibition of NF-κB by different drugs potentially alleviates SCI (Zhang et al., 2014, 2016; Ni et al., 2015). In view of this, inhibition of NF-κB could be a potential target for SCI improvement.

Resveratrol, a potent inhibitor of NF-κB, is non-toxic, inexpensive and has multiple pharmacological effects on SCI recovery (Zhao et al., 2017). Resveratrol can modulate cellular growth, autophagy, and immune response by regulating some signaling pathways such as NF-kB, SIRT1, PI3K/Akt/mTOR, ROS/Nrf2/NAF-1, and MAPK pathways (Kulkarni and Cantó, 2015; Chai et al., 2017; Cheng et al., 2018). This review aims to systematically analyze resveratrol’s role in regulating NF-κB signaling pathway in the wake of SCI.

## Pathophysiology of Sci

Secondary injury phase involves a number of pathophysiological processes including vascular changes such as hemorrhage and breakdown of BSCB, inflammation, peroxidation of lipid membranes and neural cell apoptosis (Silva et al., 2014). Spinal cord edema after SCI induces BSCB opening, leading to release of deleterious substances from blood cells such as neutrophils and macrophages, ultimately resulting in cell death as well as permanent neurological disability (Lee et al., 2012). Inflammatory response is important in SCI development. Inflammation follows immune response, which protects against pathogen. However, excessive inflammation causes damage to body. Major participatory cells of inflammation in SCI include macrophages, endothelial cells, microglia and astrocytes. Classical macrophage (M1) promotes inflammation while alternative macrophage (M2) has a contrasting effect on inflammation. M1 phenotype, activated by Th1 cytokines, LPS-induced signal transducer and activator of transcription 1 signaling, induces pro-inflammatory cytokines (TNF-α, IL-1, IL-1β, IL-6, and IL-12) and nitric oxide, leading to SCI disruption. On the contrary, Th2-induced M2 phenotype promotes anti-inflammatory cytokines (IL-10, TGF-β), contributing to attenuation of damage in injured spinal cord (Mantovani et al., 2004). Kopper and Gensel (2017) reported myelin lipid debris, a hallmark of SCI, to be a potent macrophage stimulus. Additionally, it was the target of complement-mediated clearance and inflammation, which was possibly cleared through macrophage surface receptors. Downstream results are determined by myelin-driven events that make macrophages a double-edged sword. Although macrophages are able to promote axonal growth, stem cell differentiation and tissue revascularization to facilitate repair of injured spinal cord, they (i.e., macrophages) can also contribute to SCI pathology through several mechanisms such as release of pro-inflammatory cytokine (Kopper and Gensel, 2017). Inflammation is thought to have an antithetical effect on neuroprotection in SCI. Changing process of different inflammatory factors and cells is different on temporal sequence after CNS trauma (Donnelly and Popovich, 2008). At some point during inflammatory response from destructive to constructive, drugs could improve the pathological process by interfering with inflammatory cells and their released products. In an experimental study of NSC transplantation in SCI, results evinced NSC transplantation to modulate inflammation and enhance neurological function following SCI by reducing M1 macrophages’ inflammatory activity and infiltration of neutrophils (Cheng et al., 2016).

Reactive oxygen species (ROS) and oxidative stress are important events that have been associated with SCI. Both neuronal and glia cells in injured spinal cord are sensitive to oxidative and electrophilic stress (Jia et al., 2012). Ketone metabolite, β-hydroxybutyrate, has been reported to have the capability of inhibiting class I HDACs so as to attenuate oxidative stress in SCI. Selected suppression of either HDAC1 or HDAC2 systematically mediates FOXO3a, NOX2 and NOX4 expressions (Kong et al., 2017). Regarding excitotoxicity, studies have reported glutamate receptors to mediate cell death, which was potentiated by TNF-α (Beattie et al., 2002). All these disparate mechanisms could potentially interact with each other and culminate in axonal demyelination, glial scars and neuronal diminution.

## Nf-κB Signaling Pathway Following Sci

Nuclear factor kappa-light-chain-enhancer of activated B cells is comprised of NF-κB family, inhibitor of NF-κB (IκB) and IKK. NF-κB family, also called NF-κB/Rel family, includes NF-κB1 (p50), NF-κB2 (p52), p65 (RelA), c-Rel and RelB. IκB family consists of IκBα, IκBβ and IκB𝜀 (Ahn and Aggarwal, 2005; Heise et al., 2014). IKK is a large protein complex that modulates phosphorylation of κB inhibitor. In resting cells, inactive NF-κB has been associated with inhibitor proteins, IκBα, IκBβ, or IκB𝜀, masking its nuclear localization sequence and inhibiting its DNA-binding activity (O’Dea et al., 2007). In spinal cord, NF-κB transcription factors are expressed in glial cells and blood vessels, which functions mainly in mediating various mechanisms such as inflammation.

### Exhibition of Inflammation

Inflammatory signs can be detected during acute phase of SCI as cyclooxygenase-2 (COX-2), interleukin-1β (IL-1β), interleukin-6 (IL-6) and interleukin receptors (IL-4R and IL-2Ra) are upregulated from 0.5 to 6 h following injury. Four main inflammatory cells (neutrophils, monocytes, lymphocytes and microglias) are assembled and release inflammatory cytokines. These pro-inflammatory factors, at low concentration, could trigger inflammation in order to protect normal tissues in spinal cord. When these pro-inflammatory factors are overexpressed, they tend to activate transcription factors such as NF-κB that could cause cell death, subsequently altering role of pro-inflammatory factors from positive to negative (Bareyre and Schwab, 2003). Following that, inflammation could create an environment that instigates other pathological events. Among transcription factors that are negative result of inflammation, NF-κB is of great importance. Once stimulated by either inflammatory intercellular signals or other signals like oxidative stresses such as free radicals, metabolic stress, or genotoxic stress in SCI, NF-κB signaling pathway could be induced by “classical” and “alternative” pathways.

In the “classical” pathway, NF-κB is mediated by activation of a number of cell surface receptors, including TLRs, IL-1 receptor, and TNF receptor. TLRs, a type I transmembrane proteins with extracellular domains, has a close contact relationship with NF-κB. In one of TLR signaling pathway, MyD88-dependent pathway, TLR recruits toll-interleukin 1 receptor domain containing adaptor protein at the cell membrane, facilitating recruitment of MyD88, which in turn could trigger early-phase activation of NF-κB. In another TLR signaling pathway, namely TIR-domain-containing adapter-inducing interferon-β (TRIF)-dependent pathway, TLR4 forms a signaling complex with TRIF-related adaptor molecule and TRIF, potentially culminating in late-phase activation of NF-κB, interferon regulatory factor 3 activation and interferon-β transcription (Kawai and Akira, 2010). IKK phosphorylates two N-terminal serines of IκBα and IκBβ leading to poly ubiquitination of IκBs. Then, IκBs degrade via 26S proteasome that possibly activates NF-κB (Zandi et al., 1998; O’Dea et al., 2007). Eventually, NF-κB liberated from IκBs enters the nucleus to activate target gene expression, leading to possible differential effect on gene expression.

The “alternative” pathway triggered by activation of TNF-receptor initiates NIK to stimulate IKKα-induced phosphorylation and proteolytic processing of cytoplasmic NF-κB2 precursor. Activated NF-κB2, RelB and NIK form a complex to enter the nucleus, potentially activating gene expression. This pathway is mainly involved in immune response (Brasier, 2006). Various pro-inflammatory molecules like TNF-α, IL-1, IL-6 and inducible NOS are mediated by nucleus NF-κB complex, possibly engendering inflammation in spinal cord.

Hydroxysafflor yellow A (HSYA) has anti-inflammatory and anti-oxidative effects on neuronal protection (Zhang et al., 2014). Pei et al. (2017) study evidenced effects of HSYA inhibiting NF-κB activation and curtailing TNF-α and IL-6 in spinal cord after HSYA treatment, which confirms the vital role of NF-κB in exhibition of inflammation in SCI. Also, expression of miR-372 is stimulated in cultured human NSCs by IL-1β possibly binding with NF-κB at its promoter region. Thus, miR-372 could potentially be involved in inflammatory signaling mediated by NF-κB (Zhou et al., 2017). Again, TWEAK, Fn14 and NF-κB had a synchronous alteration in a conducted study. Down-regulation of TWEAK curtailed TNF-α and IL-1β expressions. This suggests stimulation of TWEAK-Fn14 pathway might promote NF-κB expression and possibly culminate in elevated expression level of inflammatory cytokines (Xu et al., 2016).

### Free Radicals and Lipid Peroxidation

Free radicals are mainly from endoplasmic reticulum and mitochondria. Reactions between free radicals and polyunsaturated fatty acid of plasma membrane peroxidates and disrupt normal phospholipid structure of cellular biomembrane system (Jamme et al., 1995). NF-κB can be mediated by free radicals. NF-κB activation is induced by TNF-α, IL-1β, TLRs, or antigen receptor (TCR, BCR) ligation, which promotes expression of a number of genes (Gloire et al., 2006). These genes, which include mitochondrial MnSOD, are involved in anti-oxidative stress response. MnSOD is an enzyme that inhibits TNF-α-induced ROS production and cell death (Kamata et al., 2005). Inhibition of NF-κB sustains oxidative stress and stimulates either apoptosis or necrosis of cells. In contrast to ROS, induced NOS from different sources like endothelial cells and neurons trigger S-nitrosylation of JNK1 and IKKb protein kinases to impede autophagic flux in mammalian cells. S-nitrosylation of JNK1 stabilizes Beclin 1/B-cell lymphoma-2 (Bcl-2) complexes by reducing phosphorylation of Bcl-2, which can potentially block early steps of formatting autophagic membranes. S-nitrosylation of IKKb has same effect as JNK1 on Adenosine 5′-monophosphate (AMP)-activated protein kinase phosphorylation in suppressing autophagy initiation (Kaminskyy and Zhivotovsky, 2014). NF-κB, c-Fos and c-Jun expressions, as activators of oxidative response markers, suggest possible augmented production of ROS is likely to give rise to cascades of oxidative destruction. Also, lipid peroxidation and nucleic acid oxidation are elevated in injured spinal cord and motor neurons. Consequently, increased degeneration of ROS could contribute to motor neuronal death in spinal cord, which is an early causal event in the wake of SCI (Xu et al., 2005).

### Apoptosis and Demyelination of Axons

Though neuronal damage in SCI occurs predominantly through necrosis, apoptosis cannot be ignored. Intracellular hypercalcemia induced by loss of ionic homeostasis after SCI activates calcium-dependent proteases and results in mitochondria dysfunction, eventually leading to apoptotic cell death (Schanne et al., 1979). Signaling pathways such as Fas/FasL have been evidenced to mediate apoptosis. NF-κB has been reported to be involved in apoptosis. A recent study evinced that during early phase of SCI, TWEAK might assemble pro-inflammatory factors to instigate cell apoptosis through its effect on NF-κB expression (Xu et al., 2016). RIPK1 functions as a node which drives cell survival and inflammation mediated by NF-κB, caspase-8-dependent apoptotic and RIPK3/MLKL-dependent necroptotic cell death (Takahashi et al., 2014). Interplay between IKK/NF-κB and RIPK1 signaling is an important determinant of tissue homeostasis and inflammation. In the intestine and liver, NEMO regulates RIPK1 kinase activity-mediated apoptosis through NF-κB-dependent and independent functions, which are crucial for inhibiting chronic tissue injury and inflammation (Kondylis et al., 2017). A new function of TAK1 was reported to regulate checkpoint of early NF-κB-independent cell death in TNFR1 apoptotic pathway. Ub chains conjugated with RIPK1 or other TNFR1 complex I functions as scaffolds in the recruitment and activation of TAK1-binding protein 2/3–TAK1 complex and inhibition of IKK complex (NEMO-IKKa-IKKb), potentially triggering off mitogen-activated protein kinases and classical NF-κB signaling pathways that collectively promotes gene transcription, preventing cell death and sustaining inflammation. Thus, a pro-survival TNFR1 ligation will switch to a pro-apoptotic one when NF-κB response is potentially inhibited (Dondelinger et al., 2013). Besides, ROS is related to apoptosis. A previous study reported ROS generation to be required for TNF-mediated necroptosis (Vanlangenakker et al., 2011). ROS scavenging has the potentiality of inhibiting caspase activation and assembly of complex IIb on TNF stimulation (Dondelinger et al., 2013). Microglia-derived TNF-α was found to induce Puma expression, a member of BH3-only family in NPCs via an NF-κB-dependent mechanism. More specifically, NF-κB was activated in NPCs cultivated with conditioned media from activated microglia. Also, NF-κB inhibitor, BAY-117082, blocks Puma induction, and NPC apoptosis (Guadagno et al., 2013). There are reports stating taxifolin might possibly reduce neuronal apoptosis induced by cholesterol oxidation product through suppression of cell death mediated by Akt and NF-κB activation (Kim et al., 2017). In the only contrasting study, neuroprotective effect of neuronal IKK-2 in autoimmune demyelination was reported (Emmanouil et al., 2011). In rats, loss of oligodendrocytes could possibly lead to axonal demyelination and peaks at about 24 h following injury. Persistent demyelination is related to atrophy and potential death occurring in related cell bodies (Rowland et al., 2008). When demyelination happens in SCI, microglia phagocytizes myelin debris (Blank and Prinz, 2014). Degenerated myelin containing inhibitory molecules like NogoA and Oligodendrocyte-myelin glycoprotein activates FAK/PI3K/Akt/NF-κB pathway in macrophages and promotes expression of inflammatory mediators (Sun et al., 2010). Activated astrocytes and microglia potentially enhances NF-κB activation. However, there are no reports of NF-κB activation in oligodendrocytes. During remyelination, NF-κB in recruited oligodendrocyte progenitor cells is activated. These progenitor cells, which engage demyelinated axons, are differentiated into remyelinating oligodendrocytes (Blank and Prinz, 2014). A study found E6020 to induce TLR4-dependent cytokine expression such as TNFα, IL1β, IL-6 and NF-κB signaling *in vitro*. Injection of E6020 with lysolecithin into rat spinal cord white matter increased axonal sparing, accelerated myelin debris clearance, enhanced Schwann cell infiltration into demyelinated lesions and increased number of remyelinated axons (Church et al., 2017). Taking all these studies into consideration, NF-κB signaling pathway is predominately involved in various pathophysiological mechanisms of SCI, especially with regards to inflammation.

## Resveratrol Inhibits Nf-κB Signaling Pathway

**FIGURE 1:**
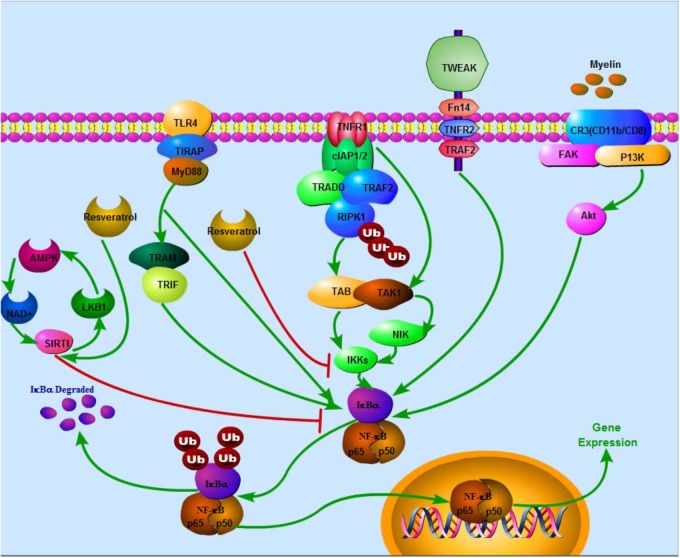
Negative effects of resveratrol on NF-κB via several pathways in SCI. In TNFR1 apoptotic pathway, Ub chains conjugated with RIPK1 function as scaffolds in the recruitment and activation of TAK1-binding protein 2/3–TAK1 complex and inhibition of IKK complex (NEMO-IKKa-IKKb), subsequently triggering off the classical NF-κB signaling pathways that collectively promote transcription of genes, which prevents cell death and sustains inflammation. In MyD88-dependent pathway, TLR recruits toll-interleukin 1 receptor domain containing adaptor protein at the cell membrane, subsequently facilitating recruitment of MyD88, which in turn triggers early-phase activation of NF-κB. Recruitment of TRAM and TRIF leads to late-phase activation of NF-κB. IKK phosphorylates IκBα and IκBβ leading to poly ubiquitination of IκBs. Degradation of IκBs results in activation of NF-κB. Besides, degenerated myelin activates FAK/PI3K/Akt/NF-κB pathway in macrophages and promotes expression of inflammatory mediators. Stimulation of TWEAK-Fn14 pathway also promotes NF-κB expression. On the contrary, SIRT1 inhibits NF-κB activation. Eventually, NF-κB enters the nucleus to activate target gene expression. Resveratrol potentially enhances SIRT1 and AMPK expressions. AMPK activation increases NAD+/NADH ratio and triggers its downstream whereas SIRT1 acts as an anti-inflammatory NAD+-dependent deacetylating enzyme via direct deacetylation of subunit of NF-κB such as p65, SIRT1 directly interacts with RelA/p65. Also, resveratrol suppresses IKK activity.

Resveratrol (*trans*-3,5,4-trihydroxystilbene), a small natural polyphenol found in red wine, grapes, peanuts and other different kinds of plant sources is widely studied and used in therapies of different diseases. Resveratrol, used in Indian herbal, traditional Chinese, and Japanese medicine for human health, can be traced back 2000 years ago, with a prime example of a well-known Indian herbal preparation being “darakchasava.” Resveratrol is a naturally polyphenolic phytoalexin first identified from *Veratrum grandiflorum* in 1939 (Takaoka, 1939) and later detected in dried roots of *Polygonum cuspidatum*, in the leaf epidermis and skin of grape berries. Resveratrol was initially applied to treat injuries (Langcake and Pryce, 1976). Cardiovascular effect of red wine was called “the French paradox,” with resveratrol attracting widespread attention in the early 1990s (Renaud and de Lorgeril, 1992). Wood et al. (2004) initially reported resveratrol to extend lifespan in yeast and in worms. Since then, numerous beneficial effects of resveratrol have been reported in different pathological conditions, experimental models and clinical studies. A growing body of evidence shows resveratrol might play potential therapeutic roles in cardiovascular disease, cancer, ischemic injury, sarcopenia, diabetes, obesity, respiratory diseases, osteoarthritis and neurodegeneration (**Table 1**). It has various pharmacological effects such as anti-oxidative, anti-inflammation, cardiovascular protection, anti-diabetic, restoration of immune system function (Lai et al., 2017), anti-apoptosis (Huang et al., 2014), anti-nociception (Pan et al., 2016), anti-depression (Finnell et al., 2016), anti-cancer activities (Chin et al., 2015; Kumar et al., 2015), neuronal protection (Nalagoni and Karnati, 2016) and regulation of metabolism (Luo et al., 2017). Biological activity of resveratrol can possibly be achieved by mediating NF-κB (Tsang et al., 2016), heme oxygenase-1 (HO-1) (Sakata et al., 2010), mitogen-activated protein kinase (MAPK) (Ma et al., 2015), endothelial nitric oxide synthase (eNOS) (Xia et al., 2010), nuclear factor E2-related factor-2 (Nfr2) (Cheng et al., 2018), estrogen receptor (ER) (Saluzzo et al., 2016) and HDAC SIRT1 (Chai et al., 2017). Based on enumerated effects, there are reports positing resveratrol could potentially be employed in the treatment of medical conditions such as Alzheimer’s disease (Moussa et al., 2017), diabetes, cardiovascular diseases as well prostate and breast cancers (Chin et al., 2015; Kumar et al., 2015; Zare Javid et al., 2017). Resveratrol functions via several signaling pathways, one being NF-κB. Accumulating studies indicate inhibition of NF-κB signaling pathway by resveratrol potentially plays a significant role in various pathological processes (**Figure 1**).

**Table 1:** Summary of beneficial effects of Resveratrol (RES).

Diseases	Species	Dose	Results	Reference
Brain injury	Rat and mouse	60 or 90 mg/kg	RES has an efficient neuroprotection against subarachnoid hemorrhage	Zhang et al., 2017
Cardiovascular disease	Rat	15 mg/kg	RES improves left ventricular function and decreases myocardial hypertrophy, fibrosis, and severity of heart failure.	Riba et al., 2017
Neurodegeneration	Mouse	10 mg/kg	RES significantly attenuates acute neurological deficits, neurodegeneration and cerebral edema after intracerebral hemorrhage	Bonsack et al., 2017
Sarcopenia	Rat	125 mg/kg	RES reduces apoptotic signaling in muscles of old animals	Bennett et al., 2013
Acute pancreatitis	Rat	10–50 mg/kg	RES attenuates pancreatic oxidative damage by down-regulating NF-κB and PI3K signaling pathways	Tsang et al., 2016
Obesity	Mouse	15 mg/kg	RES increases Cidea mRNA level and UCP1 protein expression	Arias et al., 2017
Diabetes	Mouse	40 mg/kg		
				Zhao et al., 2018
			RES attenuates testicular apoptosis in type 1 diabetic mice	
Respiratory diseases	Mouse	50 mg/kg, 40 μg/ml	RES relieves LPS-induced inhibition on SIRT1 expression and restrains activation effects of LPS on MAPKs and NF-κB activation	Ma et al., 2015
Kidney injury	Rat	0.23 μg/kg	RES inhibits inflammatory responses and improves renal function after renal Ischemia-reperfusion injury	Li et al., 2018
Colorectal cancer	Human	5 μM	RES induces apoptosis, suppresses NF-κB activation	Buhrmann et al., 2018
Thyroid cancer	Rat	100 μM	RES inhibits NF-κB/p65 signaling, IL-6 and COX-2 expressions	Zheng et al., 2018
Breast cancer	Human	0–100 μM	RES inhibits breast cancer cellular proliferation	Poschner et al., 2018
Pancreatic cancer	Human	50 μM	RES promotes pancreatic cancer apoptosis through ROS/Nrf2/NAF-1 pathway	Cheng et al., 2018
ovarian cancer	Human	10, 20, or 30 μM	RES reduces cell growth and metabolism of SKOV-3 aggregates	Tino et al., 2016
Liver cancer	Human	100 μM	RES inhibits PI3K/AKT pathway by SIRT1 activation	Chai et al., 2017
Gastric cancer	Human	100 μM	RES inhibits growth of MGC-803 cells by inhibiting Wnt signaling pathway	Dai et al., 2018
Prostate cancer	Mouse	625 mg/kg		
				Harper et al., 2007
			RES suppresses prostate cancer progression	
Osteoarthritis	Rat	50 mg/kg	RES reduces inflammatory responses by inhibiting NF-κB expression	Wei et al., 2018

Reports evidence resveratrol to suppress various cytokines such as TGFβ, MMP, COX-2, TNF-α, IL-1β, IL-6 and intercellular cell adhesion molecule-1 (ICAM-1). All these demonstrate the linkage between resveratrol and NF-κB owing to expression of these cytokines being mediated by NF-κB signaling pathway. In liver diseases, prevention of fibrosis by resveratrol is concomitant with preventing translocation of NF-κB and down-regulating profibrotic cytokine TGFβ (Chávez et al., 2008). In a rat model of early polymicrobial sepsis-induced acute kidney injury, resveratrol significantly improved tubular epithelial cell injury and renal function, subsequently enhancing rat survival rate. This was concomitant with substantial decrement of TNF-α, IL-1β and IL-6 expressions in serum content and renal mRNA (Wang et al., 2017). Resveratrol reduces ICAM-1 expression in attenuation of endothelial inflammation, which is partly mediated through miR-221/222/AMPK/p38/NF-κB pathway (Liu et al., 2017). In human fibroblast-like synoviocytes, resveratrol was reported to inhibit COX-2/PGE expression so as to suppress particulate matter-induced inflammatory signaling pathways (Tsai et al., 2017). Moreover, resveratrol acts on two critical transcription factors to downregulate COX-2 expression: p50/p65 under the control of signaling cascade IKKBα/IκBα and activator protein-1 complex of MAPK/ERK/p38/JNK (Bhat and Pezzuto, 2002; Kundu et al., 2006; Cianciulli et al., 2012). In ovarian cancer cell aggregates, resveratrol and acetyl-resveratrol potentially inhibits cell growth, which is correlated with decreased secretion of vascular endothelial growth factor and attenuation of NF-κB (Tino et al., 2016). As an NF-κB signal transduction inhibitor, resveratrol inhibits MMP-9 and COX-2 so as to prevent disruption of blood brain barrier during neuroinflammation in carcinogen-induced brain endothelial cells (Annabi et al., 2012).

Inhibition of p65 subunit translocation by resveratrol to the nucleus is a mechanism that was deduced from an experimental hepatocarcinogenesis model (Bishayee et al., 2010). Resveratrol decreases IκB phosphorylation as well as phosphorylation, acetylation and translocation of NF-κB p65 induced by TNF-α (Liu et al., 2017). Reduction of IκB phosphorylation, p65 protein levels in nucleus and NF-κB transcriptional activity by resveratrol is involved in resveratrol-induced apoptosis (Li et al., 2013). Resveratrol can potentially regulate levels of NF-κB-mediated miRNAs. Resveratrol inhibits miR-21 expression to suppress NF-κB activity (Li et al., 2013). Resveratrol was reported to downregulate miR-21 expression and other comiRs including miR-30a-5p, miR-19 as well as their targeted or related components (Wang et al., 2015). Interestingly, in U251 cells, over-expression of miR-21 reversed resveratrol on NF-κB activity and apoptosis (Li et al., 2013).

Also, resveratrol requires the enzyme, SIRT1, so as to inhibit upregulation of NF-κB-regulated gene products in promoting chondrogenic differentiation of mesenchymal stem cells (Buhrmann et al., 2014).

## Resveratrol’s Stimulation of Sci Amelioration Is Correlated to Nf-κB Signaling Pathway Inhibition

Though resveratrol has multiple beneficial health effects such as anti-inflammation and anti-apoptosis, some of these effects on SCI protection still remain unclear. Resveratrol significantly exerts effect on preserving structure and morphology of damaged tissues, protecting neurons from traumatic injury-induced apoptosis and promoting locomotive activity of hindlimb motor neurons in rats following SCI, which is possibly related to its pleiotropic effects including anti-oxidation, anti-inflammation and anti-apoptosis. Resveratrol improves recovery of injured tissues in spinal cord via mediating participatory cytokines. Regarding anti-oxidation, resveratrol can reversely increase superoxide dismutase and decrease malondialdehyde to inhibit oxidation in SCI. Thus, resveratrol can potentially accelerate scavenging and protect cellular structure from damage to inhibit overproduction of free radicals. Concerning anti-inflammation, resveratrol can potentially curtail injury-induced myeloperoxidase and other inflammatory factors such as IL-10 and TNF-α and relieve neutrophil infiltration to inhibit inflammation cascade. On the subject of anti-apoptosis, resveratrol could elevate expression of Bcl-2, the anti-apoptotic substrate while inhibiting Bax and caspase-3, which attenuates triggering of apoptosis and reducing neuronal death (Liu et al., 2011).

Also, resveratrol could mediate SIRT1-AMPK signaling pathway to regulate autophagy and apoptosis that possibly plays a neuroprotective role in SCI. Resveratrol was found to enhance SIRT1 and AMPK expressions. Apoptotic expression is significantly suppressed by resveratrol on protein and mRNA level, which could possibly be related to the up-regulation of SIRT1/AMPK signaling pathway in the wake of SCI. Both SIRT1 and AMPK are crucial substrates in mediating autophagy (Zhao et al., 2017). AMPK activation increases NAD+/NADH ratio and triggers its downstream whereas SIRT1 acts as an anti-inflammatory NAD+-dependent deacetylating enzyme via direct deacetylation of subunit of NF-κB such as p65 (Yeung et al., 2004; Yang et al., 2012). SIRT1 directly interacts with RelA/p65 via deacetylation of RelA/p65 on lysine 310, a site crucial for NF-κB transcriptional activity, to inhibit NF-κB transcription. Employment of resveratrol potentiates SIRT1 protein on cIAP-2 gene, an effect that possibly correlates with liberating cIAP-2 gene from regulation of NF-κB and sensitizing cells to TNFα-induced apoptosis (Yeung et al., 2004). Besides, SIRT1 can potentially inversely activate AMPK or induce phosphorylation of peroxisome proliferators activated receptor-γ coactivator-1α, thereby inhibiting RelA/p65-mediated NF-κB signaling. AMPK has been evinced to be a negative regulator of NF-κB signaling and is involved in inflammatory response in macrophages (Racioppi et al., 2012; O’Neill and Hardie, 2013). These negative effects by SIRT1-AMPK signaling pathway on NF-κB activity leads to enhanced IL-12 production and anti-inflammation (Liu et al., 2016).

There is the possibility of resveratrol inhibiting NF-κB activity via SIRT1-AMPK signaling pathway to exert its beneficial effects on improving SCI. Hence, SIRT1-AMPK signaling pathway can be a possible bridge connecting autophagy and inflammation. Studies have indicated polydatin, a glucoside of resveratrol, to possess potent antioxidative effects. It has the potentiality of partly solving the problem of poor viability of transplanted bone marrow stem cells (BMSCs), which limits therapeutic efficacy. Thus, polydatin with BMSCs could possibly improve therapeutic effect of SCI (Chen et al., 2016). Resveratrol attenuates SCI-induced inflammatory damage in rats’ lungs, which was accompanied by curtailment of pro-inflammatory factors, up-regulation of SIRT1 and anti-inflammatory cytokines as well as suppression of NF-κB activity (Liu et al., 2015). Resveratrol was also demonstrated to exert some effect on enervated sub-lesional bone loss that is associated with repressed oxidative stress, curtailed inflammation, depressed peroxisome proliferators-activated receptor γ signaling and restored Wnt/β-catenin and insulin-like growth factors-1 signaling in SCI rats (Wang et al., 2013).

## Conclusion

Nuclear factor kappa-light-chain-enhancer of activated B cells signaling pathway plays a crucial role in exhibition of inflammation, promotion of damage caused by free radicals and lipid peroxidation as well as facilitation of apoptosis and axonal demyelination in SCI. Resveratrol has been studied in the treatment of various diseases in different organs via NF-κB signaling pathway. Beneficial effect of resveratrol in SCI is explicated as evidenced previously. Thus, we hypothesize resveratrol to potentially improve SCI by inhibiting NF-κB signaling pathway. With that said, more direct researches are needed to explore interior mechanisms between resveratrol and NF-κB signaling pathway following SCI, which in turn could support promising value of resveratrol in SCI therapy. Finally, appropriate and precise phase at which resveratrol can be employed needs to be elucidated.

## Author Contributions

XL participated in the study design. LX, JZ, SZ, and XL prepared the first draft of the manuscript. LX, BB, and XL participated in revision of the manuscript and approved the final paper.

## Conflict of Interest Statement

The authors declare that the research was conducted in the absence of any commercial or financial relationships that could be construed as a potential conflict of interest.

## Abbreviations

Bcl-2: B-cell lymphoma-2
BSCB: blood-spinal cord barrier
COX: cyclooxygenase
ERK: extracellular signal-regulated protein kinase
FAK: focal adhesion kinases
Fn14: fibroblast growth factor-inducible 14
HDAC: histone deacetylase
HSYA: hydroxysafflor yellow A
IKK: IκB kinase
IL: interleukin
JNK: c-Jun N-terminal kinase
miR-372: microRNA-372
MLKL: mixed lineage kinase domain-like
MMP: metalloproteinase
MnSOD: Mn-superoxide dismutase
MyD88: myeloid differentiation primary response gene 88
NEMO: NF-κB essential modulator
NF-κB: nuclear factor kappa-light-chain-enhancer of activated B cells
NIK: NF-κB inducing kinase
NOS: nitric oxide synthase
NPCs: neural precursor cells
NSC: neural stem cell
PI3K: phosphoinositide 3-kinase
RIPK1: receptor-interacting protein kinase 1
ROS: reactive oxygen species
SCI: spinal cord injury
SIRT1: Sirtuin 1
TAK1: TGF-b-activated kinase 1
TGFβ: transforming growth factor-β
TLRs: Toll-like receptors
TNFR1: tumor necrosis factor receptor 1
TWEAK: TNF-like weak inducer of apoptosis.
